# Single-Molecule Approach to 16S rRNA for Vaginal Microbiome Signatures in Response to Metronidazole Treatment

**DOI:** 10.1128/spectrum.01706-22

**Published:** 2023-05-18

**Authors:** Hanyu Qin, Jiao Jiao, Disi A, Mingxi Hua, Kai Han, Haonan Du, Zhen Wang, Jiarui Li, Dai Zhang, Bingbing Xiao, Chen Chen

**Affiliations:** a Department of Obstetrics and Gynecology, Peking University First Hospital, Beijing, China; b Biomedical Innovation Center, Beijing Shijitan Hospital, Capital Medical University, Beijing, China; c Center of Reproductive Medicine, Shengjing Hospital of China Medical University, Shenyang, China; University of Utah and ARUP Laboratories

**Keywords:** single-molecule sequencing approach, *Lactobacillus*, *Prevotella*, bacterial vaginosis, metronidazole, vaginal microbiology, 16S rRNA gene

## Abstract

Bacterial vaginosis (BV) is the most common infection of the lower reproductive tract among women of reproductive age, characterized by a depletion of health-associated *Lactobacillus* and an overgrowth of anaerobes. Metronidazole has been recommended as a first-line therapy for treating BV for decades. Although most cases are cured by the treatment, recurrent infections of BV seriously affect women’s reproductive health. Until now, limited information on the vaginal microbiota has been explored at the species level. Here, we adopted a single molecular sequencing approach for the 16S rRNA gene, named FLAST (full-length assembly sequencing technology), to analyze the human vaginal microbiota that improved species-level resolution for taxonomy and identified microbiota alterations in the vaginal tract in response to treatment with metronidazole. Appling high-throughput sequencing, we identified 96 and 189 novel full-length 16S rRNA gene sequences in *Lactobacillus* and *Prevotella*, respectively, which had not previously been reported in vaginal samples. Moreover, we found that Lactobacillus iners was significantly enriched in the cured group before metronidazole treatment, and that was maintained in a high frequency after the treatment, suggesting an important role for this species in response to metronidazole treatment. Our research also highlights the importance of the single-molecule paradigm for progressing the field of microbiology and applying these insights to better understand the dynamic microbiota during BV treatment. Subsequent novel treatment approaches should be proposed to improve BV treatment outcomes, optimize the vaginal microbiome, and reduce gynecological and obstetric sequelae.

**IMPORTANCE** Bacterial vaginosis (BV) is a common infectious disease of the reproductive tract. Metronidazole treatment, as the first line of treatment, frequently fails at recovery of the microbiome. However, the precise types of *Lactobacillus* and other bacteria involved in BV remain unclear, and this has resulted in a failure to identify potential markers to predict clinic outcomes. In this study, we adopted a 16S rRNA gene full-length assembly sequencing technology for the taxonomy analysis and evaluation of vaginal microbiota before and after treatment with metronidazole. We additionally identified 96 and 189 novel 16S rRNA gene sequences in *Lactobacillus* and *Prevotella* species, respectively, in vaginal samples, which improves our understanding of the vaginal microbiota. Moreover, we found that the abundance of Lactobacillus iners and Prevotella bivia before treatment was associated with a lack of cure. These potential biomarkers will help to facilitate future studies aimed at improving BV treatment outcomes, optimize the vaginal microbiome, and reduce adverse sexual and reproductive outcomes.

## INTRODUCTION

Bacterial vaginosis (BV) is the most common type of vaginal dysbiosis, and rates among women globally vary widely, affecting up to 58% of women worldwide; recent estimates suggest a 30% prevalence in the United States, while rates in sub-Saharan Africa may exceed 50% (1, 2). Rates are generally higher in black and Hispanic populations and lower in white and Asian populations (2, 3). BV is often caused by a loss of health-associated *Lactobacillus* species and replacement by nonoptimal bacteria, increasing species diversity and abundance in the vagina (4–6), which can induce inflammatory disease (7), miscarriage, preterm labor, postpartum endometritis, and acquisition of sexually transmitted infections (8, 9).

According to the recommendation of the Centers for Disease Control and Prevention, metronidazole is used as the first-line therapeutic drug for women with BV, but it does not cure all cases, and there is a very high rate of recurrence (approximately 60% in the United States) (10). The current 16s rRNA gene sequencing method has limited recognition of the changes of the vaginal microbiome before and after metronidazole treatment (11–14). We intend to explore the new method to learn more about the response of the vaginal microbiome to metronidazole so as to facilitate the use of a new targeted therapy for bacteria that are not effected by metronidazole treatment and improve the cure rate of BV in the future.

Sequencing-based taxonomic analysis of the *Lactobacillus* genus complex (LGC) highlights the importance of typing of *Lactobacillus* in BV treatments (15). Using taxonomic analysis, the behavior of different LGC organisms might be used to predict infection risks and predisposing health conditions or medical treatments (15). In addition, recent research has uncovered additional *Lactobacillus* species in ecological niches, especially in the human vaginal tract (16, 17). However, most of these processes are based on partial 16S rRNA gene sequences, which has resulted in some *Lactobacillus* being erroneously assigned to various species (18). If *Lactobacillus* is misclassified, there is a bias in observation that Lactobacillus to the changes in the vaginal microbiota before and after metronidazole treatment (19). In this study, we adopted a 16S rRNA gene full-length assembly sequencing technology (i.e., FLAST) for the taxonomic analysis and evaluation of the vaginal microbiota before and after treatment with metronidazole. The advantages of this new approach to generating novel complete *Lactobacillus* and *Prevotella* 16S rRNA gene sequences include the provision of a more complete data set for further 16S rRNA gene vaginal tract surveys. Further, comparative analysis of *Lactobacillus* and *Prevotella* revealed substantial intraspecies variation in the 16S rRNA gene and facilitated high discrimination of changes in the vaginal microbiota in response to metronidazole treatment.

## RESULTS

### Identification of unique species by full-length 16S rRNA gene sequences.

In this study, we enrolled a total of 46 women who met the BV diagnostic criteria (Table 1). After all of these patients received the standard 5-day metronidazole treatment, 20 patients were cured, while the remaining 26 patients were uncured (Table 1). For uncured patients with complementary therapy, we identified 6/20 (30.0%) cured patients and 8/26 (30.8%) uncured patients who had been diagnosed with BV with a Nugent score of 4 to 10 (see Table S1 in the supplemental material).

**TABLE 1:** Demographic characteristics and therapeutic outcomes of study subjects before treatment^*a*^

Characteristic or outcome	Cured group	Uncured group	*P* value
No. of samples	20	26	
Average age (yrs) (range)	38 (33–39)	38 (32–44)	0.52
BV diagnosis and treatment of metronidazole			
Before treatment			
Average Nugent score (range)	8 (8–8)	8 (8–8)	0.49
Average pH (range)	4.8 (4.6–5.0)	4.8 (4.6–5.0)	0.67
After treatment			
Average Nugent score (range)	1 (0–1)	7 (4–8)	<0.0001
Average pH (range)	4.1 (3.8–4.1)	4.7 (4.6–4.8)	0.01

a All patients were diagnosed with BV by Nugent score and treated with metronidazole.

Metagenomic 16S rRNA gene sequences were assembled and clustered into 1,103 operational taxonomic units (OTUs) with 183 OTUs that had not been annotated to any species previously. Among the remaining 920 OTUs, 102 OTUs were annotated to the *Lactobacillus* phylogenetic lineages and 215 OTUs were annotated to the *Prevotella* phylogenetic lineages. Compared to previously published metagenomic studies (20), our data set provides the first large-scale full-length 16S rRNA gene data set that might increase the discrimination of the taxonomy of species that commonly exist in the human vagina.

### Analyses of the vaginal microbiota in samples collected from patients treated with metronidazole.

**FIG 1:**
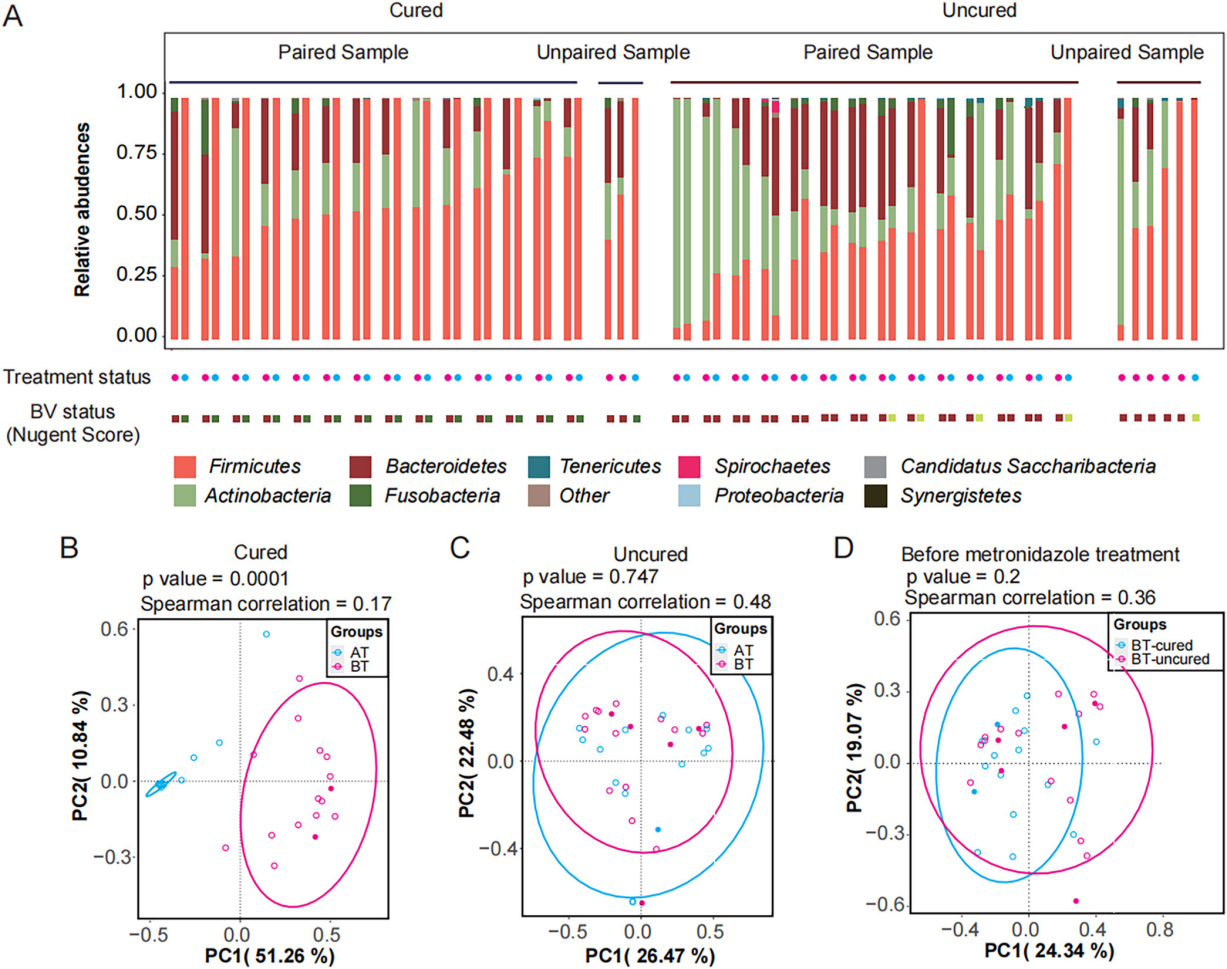
Full-length amplicon sequencing (FLAST) of 16S rRNA genes in vaginal microbiota from patients with BV before and after treatment with metronidazole. (A) Dynamic changes of microbial compositions in the top 10 phyla. The treatment status is marked under the bar plot, where pink and blue dots represent time points of BT and AT, respectively. BV status was evaluated using the Nugent score with green (0 to 3), yellow (4 to 6), and brown (7 to 10). “Other” represents genera other than the top 14 in abundance. (B to D) Principal-coordinate analysis revealing the differences: AT and BT in the cured group at the species-level (B), AT and BT in the uncured group (C), and BT in the cured and uncured groups (D). PERMANOVA of the Bray-Curtis distance was used to compute *P* values to assess the significant difference between the two groups. Correlations between groups were also computed by Spearman correlation.

Theoretically, FLAST provides full-length 16S rRNA gene sequencing, which has increased the discrimination of 16S rRNA gene data (20). We identified changes in the composition of the vaginal microbiota among four groups categorized according to whether samples were taken before or after metronidazole treatment (BT and AT groups, respectively) and from cured and uncured patients (Fig. 1A). Before treatment, all patients displayed dysbiosis in the vaginal microbial composition, characterized by low levels of *Firmicutes* and an abundant presence of *Actinobacteria* and *Bacteroidetes* (Fig. 1A). After treatment, *Firmicutes* was the dominant bacterium, and the abundance of other anaerobic bacteria decreased in cured patients (Fig. 1A). We also performed principal-coordinate analysis (PCoA) with Bray Curtis distances describing the microbial composition and abundance (Fig. 1B to D). The vaginal microbiota composition at the species level in the cured group post-metronidazole treatment was distinct from microbiota composition prior to treatment (Fig. 1B, *P* < 0.05). Differences in the vaginal microbiome composition in uncured BV patients have not been observed between pre- and posttreatment samples at the species level (Fig. 1C, *P* > 0.05). Again, we compared the composition of the vaginal microbiota at the species level before treatment between the cured and uncured groups and found that there were no significant differences (Fig. 1D, *P* > 0.05). The same results can be observed at the phylum level (Fig. S1A, *P* < 0.05; Fig. S1B and C, *P* > 0.05) and genus level (Fig. S1D, *P* < 0.05; Fig. E and F, *P* > 0.05).

It is important to identify the potential markers in the microbiota to predict clinical outcomes of metronidazole treatment for BV patients (21, 22). Therefore, we calculated the correlation of the cured and uncured groups before treatment. The data clearly highlighted that the correlation between the cured and uncured groups before treatment at the phylum level (Fig. S1C, correlation *=* 0.75) is higher than that at the species level (Fig. 1D, correlation = 0.36) and genus level (Fig. S1F, correlation = 0.50). This suggested that increased discrimination of species might be an efficient approach to monitor changes in the vaginal microbiota; such changes may be predictive of different outcomes before treatment and help identify potential markers to predict clinical outcomes.

### Identification of metronidazole treatment genus- and species-specific taxa.

**FIG 2:**
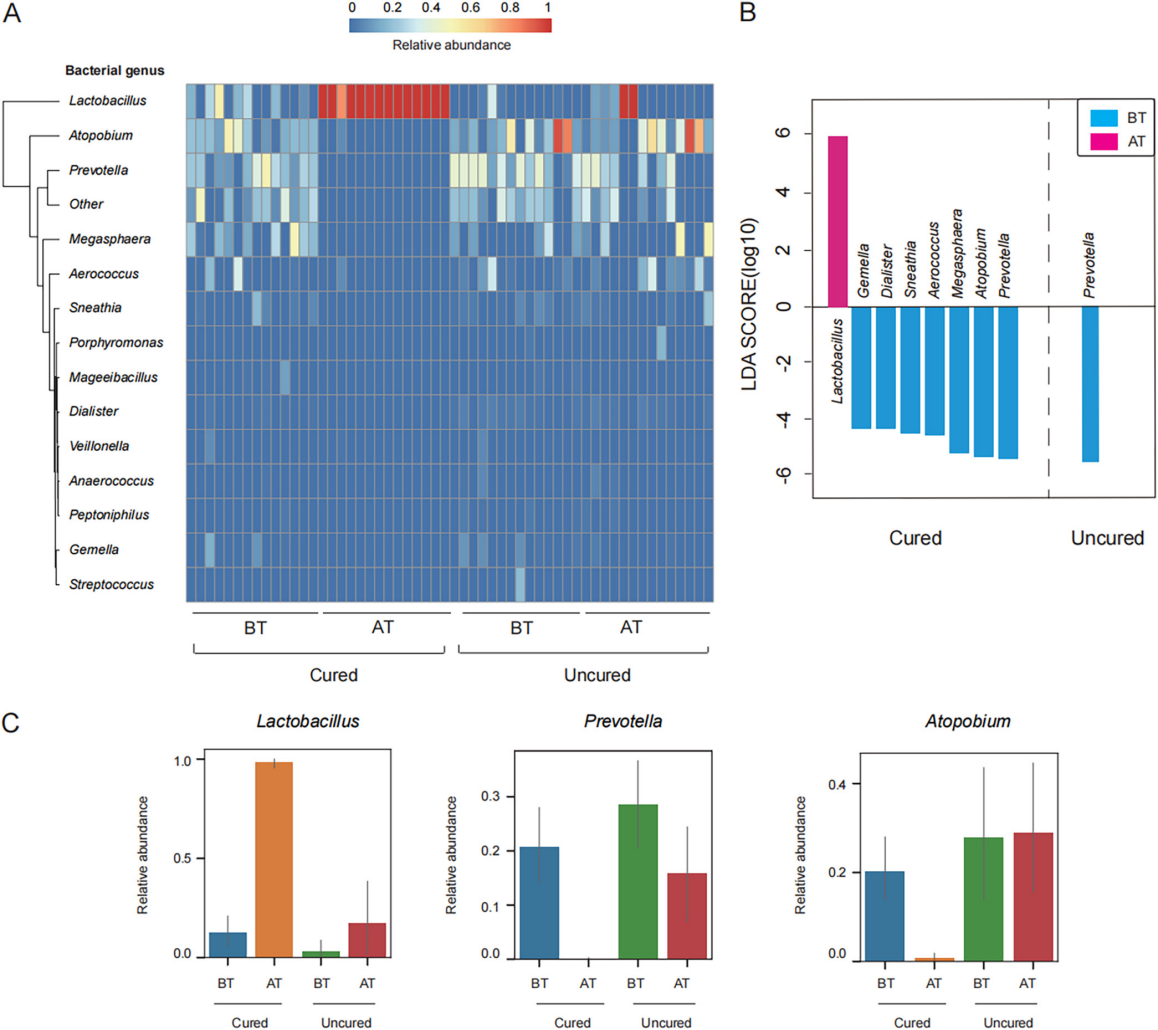
Taxonomic diversity of cured and uncured patient samples in the genus determined by FLAST. (A) Heatmap showing the relative abundance (range, 0 to 1) of the top 15 genera in all paired samples. The species-level heatmap is described in Fig. S2A. (B). Significantly differentially distributed genera (LDA score, >4) between BT and AT in the uncured patients and between BT and AT in the uncured group. (C) Relative abundances of *Lactobacillus*, *Prevotella*, and *Atopobium*. Each bar shows the mean value, and error bars show 95% confidence intervals.

To identify the impact of metronidazole treatment, we compared the abundance of organisms in the cured group BT and AT and in the uncured group BT and AT at the genus level using linear discriminant analysis effect size (LEfSe). In the cured group, we found that the abundance of *Lactobacillus* increased after treatment (Fig. 2A and B). Further, the proportion of *Prevotella*, *Atopobium*, and *Megasphaera* significantly decreased after treatment in the cured group (Fig. 2B). *Gardnerella* genus sequences were not detected in our study. Additional sequence analysis displayed that three mutations were observed between common primer 27F and the reference *Gardnerella* strain (Fig. S2), whereas they were not observed in other strains. The mismatch position on primer 27F would be responsible for the inhibition of the PCR amplification process, which subsequently failed to detect *Gardnerella*. However, we did not observe any mutations in other major species, including Lactobacillus iners (AEXK01000007), *Atopobium* (ACGK02000001), Prevotella bivia (AB547673), and *Megasphaera* (FMFF01000017), in vaginal samples (Fig. S2). In the uncured group, most genera were unchanged, and only *Prevotella* abundances changed significantly (Fig. 2B). The changes of *Lactobacillus*, *Prevotella*, and *Atopobium* were indicated by the detailed proportions in the four groups (Fig. 2C).

Next, we compared species-specific taxonomies. Heatmaps illustrating the abundance of bacterial taxa clearly showed that the dominant species was *L. iners* in the cured group posttreatment (Fig. S3A and B) and showed decreasing abundances of Atopobium vaginae, *Megasphaera* spp., and Prevotella timonensis in the cured groups after treatment (Fig. S3B). Moreover, we observed a significant downregulation only in Tissierellia bacterium KA00581 in the uncured group posttreatment (Fig. S3B). Importantly, comparison of the detected species-specific taxa also revealed differences in the abundance of *L. iners*, *A. vaginae*, *P. timonensis*, and *T. bacterium* KA00581 among the four groups (Fig. S3C). Therefore, we confirm that *L. iners* is not sensitive to metronidazole treatment and is associated with a rapid recovery of *Lactobacillus* in the vagina.

### Identification of molecular functions in response to metronidazole treatment.

To identify the predicted function of the response to metronidazole treatment, we compared the composition of functional elements in the four groups (BT and AT groups in cured and uncured patients), and analyzed the functional differences among the groups. The metabolic pathway of the microbial community was determined using MetaCyc as a reference database.

As the microbial composition in response to metronidazole treatment in cured patients differs from that in uncured patients, the function of microbial communities was consistently changed. Following metronidazole treatment in the cured group, we found that the functions of the polyisoprenoid biosynthesis (Escherichia coli), acetylene degradation, superpathway of geranylgeranyl diphosphate biosynthesis I (via mevalonate), mevalonate pathway I, and lactose and galactose degradation I were significantly increased. In contrast, the Calvin-Benson-Bassham cycle, tRNA charging, the pentose phosphate pathway (nonoxidative branch), inosine-5′-phosphate biosynthesis I, and 5-aminoimidazole ribonucleotide biosynthesis I were significantly decreased (Fig. S4A). These changes in metabolic functions were not observed in uncured patients when we compared the uncured BT and AT groups (Fig. S4B).

Differences in metabolic function can also provide potential markers to predict the clinical outcomes of metronidazole treatment. To further explore this, we compared the microbial functional profiles between cured and uncured patients before treatment (Fig. S4C). We found that UMP biosynthesis and glycolysis II (from fructose 6-phosphate) significantly increased. Moreover, *S*-adenosyl-l-methionine cycle I, starch degradation V, superpathway of 5-aminoimidazole ribonucleotide biosynthesis, 5-aminoimidazole ribonucleotide biosynthesis II, and 5-aminoimidazole ribonucleotide biosynthesis I significantly decreased in cured patients (Fig. S4C). These differentially expressed pathways provide more detailed clues for the prediction of clinical outcomes before the metronidazole treatment.

### Identification of novel *Lactobacillus* in vaginal samples and alternations in their composition following metronidazole treatment.

To identify the novel *Lactobacillus* and trace alternation of *Lactobacillus* in response to metronidazole treatment, we compared the complete 16S rRNA gene sequence to the consensus sequences that were obtained by multiple sequence alignment of all 16S rRNA gene sequences in this study.

**FIG 3:**
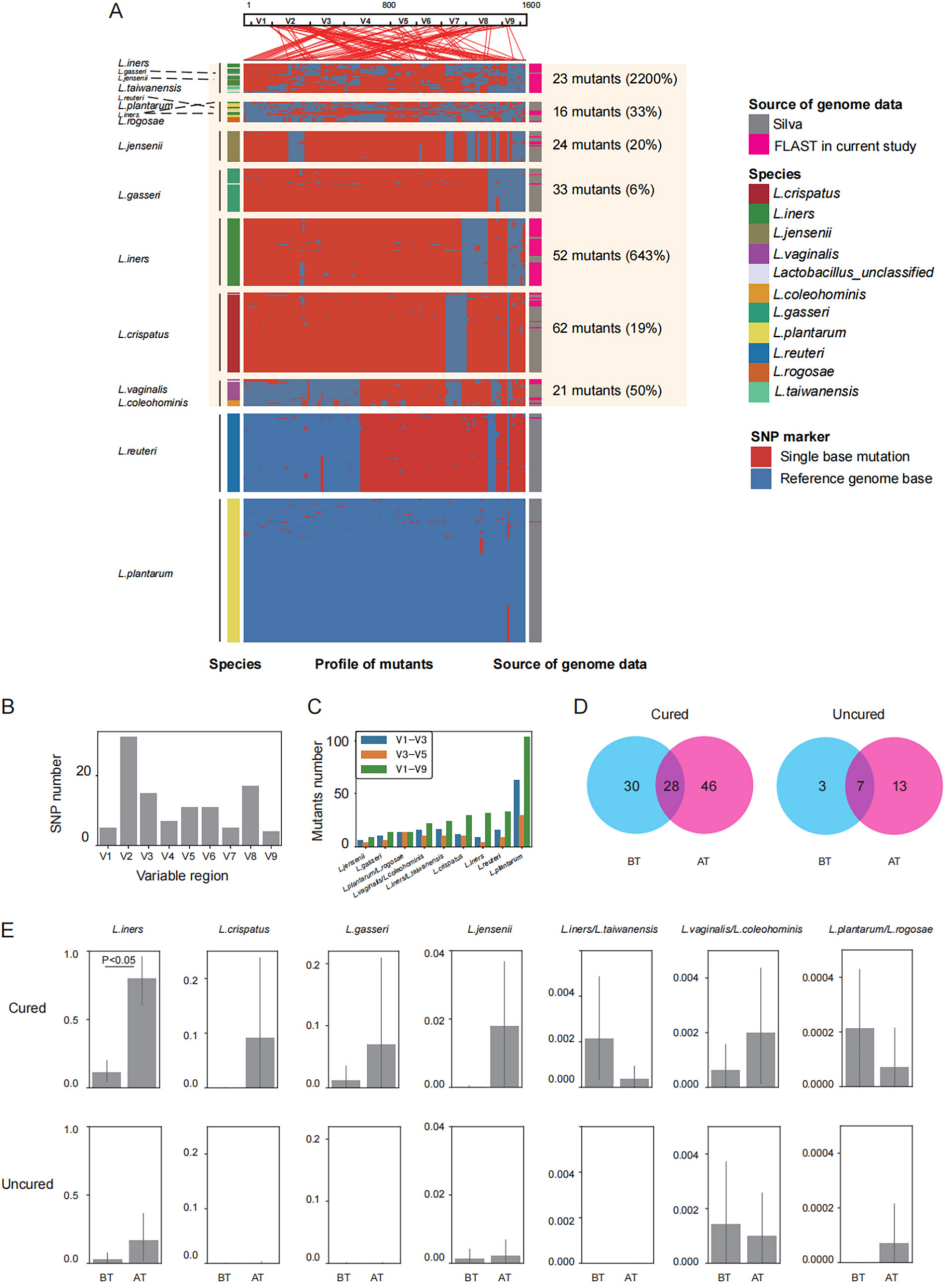
Identification of *Lactobacillus* intraspecies variation in 16S rRNA genes by FLAST. (A) Intraspecies variations in *Lactobacillus* 16S rRNA genes. V1 to V9 at the top show the 16S rRNA gene 5′ to 3′. From left to right, we marked the species annotated using mothur, mutations, and sources of genome data. Intraspecies variations are clustered according to a presence/absence matrix (using pheatmap in R). The positions with SNPs in 16S rRNA genes are indexed by lines in coordinate genome sites. Complexes are named by the combination of the top 2 species or the species that account more than 10% and constitute the most sequences. According to the distances of presence/absence matrix, *Lactobacillus* species were categorized into 9 lineages and are separated with gray lines. (B) SNP numbers observed in each variable region of 16S rRNA gene. (C) Comparison of mutation numbers observed using different 16S rRNA gene loci. V1 to V3 represent data using only variable regions from V1 to V3. V3 to V5 represent data using only three variable regions from V3 to V5. These two genomic sequences are commonly used in 16S rRNA gene detection. V1 to V9 represents data using the full length of 16S rRNA gene sequences in this study. (D) Distribution of mutants of *Lactobacillus* species in the cured group BT versus AT and the uncured group BT versus AT. (E) Relative abundances of six *Lactobacillus* lineages presented as bar plots. *P* values were calculated with the Wilcox test to assess the significance of the relative abundance in response to metronidazole between the BT and AT groups. Each bar shows the mean value, and error bars show 95% confidence intervals.

These genetic variations were widely dispersed in the reference genes and present as 701 unique *Lactobacillus* 16S rRNA gene OTUs (Fig. 3A and Table S2). These 16S rRNA gene sequences have at least 1% genetic difference, and most occurred in variable region 2 (V2) with 31 single nucleotide polymorphism (SNP) sites (Fig. 3B). V1 to V3 and V3 to V5 are two major regions used for amplicon metagenomic sequencing to determine species abundance. Compared to these approaches, the full-length 16S rRNA gene data highlighted 265 mutants in *Lactobacillus*, while only 154 and 94 mutants were observed in V1 to V3 and V3 to V5, respectively (Fig. 3C). The increased discrimination displayed various levels in different *Lactobacillus* species.

According to above-described SNP matrix, OTUs in *Lactobacillus* were clustered as nine genetic lineages, *L. iners* complex (L. taiwanensis and *L. iners*), L. plantarum (*L. plantarum* and L. rogosae), L. jensenii, L. gasseri, *L. iners*, L. crispatus, L. vaginalis (*L. vaginalis* and L. coleohominis), L. reuteri, and *L. plantarum* (Fig. 3A). Despite there being few reports of two of the lineages (L. reuteri and *L. plantarum*) in the vagina (23, 24), the first 7 lineages were previously often reported as occurring in the human vagina and were widely thought to be associated with the vaginal microbiota (25, 26). In the study, we identified an additional 2,200%, 33%, 20%, 6%, 643%, 19%, and 50% novel sequence/reference sequence in these 7 lineages, which may be considered new reference sequences for the present database (Fig. 3A).

The increased discrimination for *Lactobacillus* also increased our ability to determine whether the mutants changed during the metronidazole treatment. The distribution of several mutants contained in *Lactobacillus* species was different in the cured (before treatment versus after treatment, BT versus AT) and uncured group (BT versus AT) groups (Fig. 3D). The BT and AT groups in the cured patients shared 28 strains, but with 30 strains specific before treatment and 46 strains specific after treatment. In contrast, there were only 7 shared mutants, with 3 specific mutants pretreatment and 13 specific mutants posttreatment in the uncured group (Fig. 3D). The higher proportions of *Lactobacillus* in the cured group suggested that more protective *Lactobacillus* might have existed in these patients before the treatment, and *Lactobacillus* maintained and persisted after the metronidazole treatment. However, a low proportion of persisted *Lactobacillus* was observed in the uncured patients. The varied ability of *Lactobacillus* to resist metronidazole might be another key factor in the effects of metronidazole treatment.

To further investigate the impact of metronidazole on *Lactobacillus* species, we compared the abundance of *Lactobacillus* species before and after metronidazole treatment in cured and uncured patients (Fig. 3E). With the increasing level of discrimination of *Lactobacillus* species, our data displayed that the abundance of *L. iners* was significantly upregulated after treatment (Fig. 3E, *P* < 0.05), as well as other dominant *Lactobacillus* species (including L. crispatus, L. gasseri, and L. jensenii; abundance, >1%) (Fig. 3E). We also found that L. jensenii is also correlated with *L. iners* (Fig. S5A). These data suggest that metronidazole treatment might at least specifically increase the abundance of *L. iners*, which could be considered a potential marker for metronidazole treatment.

### Identification of novel *Prevotella* species in vaginal samples and changes in their composition following metronidazole treatment.

**FIG 4:**
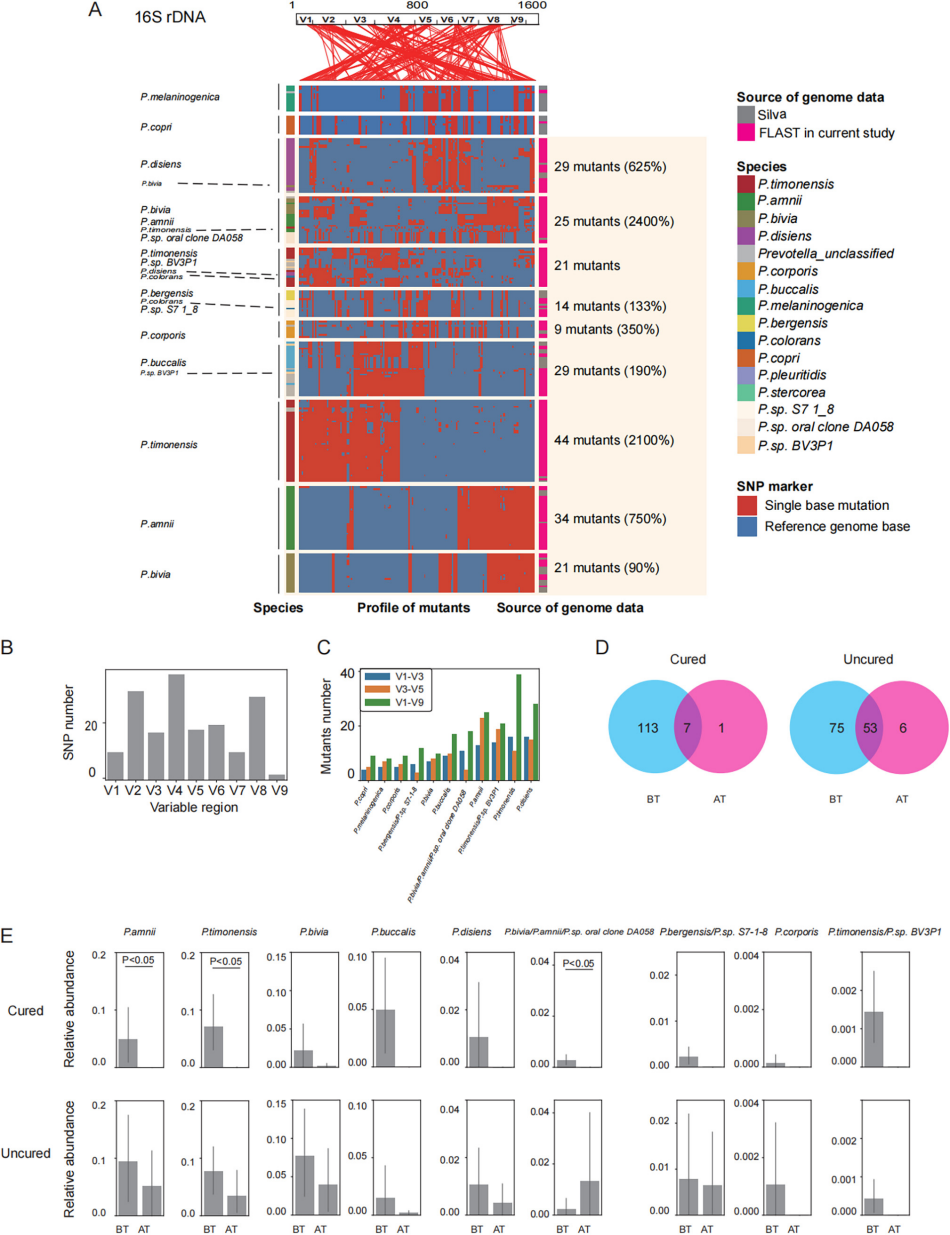
Identification of *Prevotella* intraspecies variation in 16S rRNA genes using FLAST. (A) Intraspecies variations in *Prevotella* 16S rRNA genes. V1 to V9 at the top refer to the 16S rRNA gene 5′ to 3′. From left to right, we marked the species annotated using mothur, mutations, and the sources of genome data. Intraspecies variations are clustered according to a presence/absence matrix (using pheatmap in R). The positions with SNPs in the 16S rRNA gene are indexed by lines in coordinate genome sites. Complexes are named by the combination of the top 2 species or the species that accounted more than 10% and constitute the most sequences. According to the distances of the presence/absence matrix, *Prevotella* species were categorized into 11 lineages and are separated with gray lines. (B) SNP numbers observed in each variable region of the 16S rRNA gene. (C) Comparison of mutation numbers observed using different 16S rRNA gene loci. V1 to V3 represent data using only variable regions from V1 to V3. V3 to V5 represent data using only three variable regions from V3 to V5. These two genomic sequences are commonly used in 16S rRNA gene detection. V1 to V9 represent data using the full length of the 16S rRNA gene sequence in this study. (D) Distribution of mutants of *Prevotella* in the cured group BT versus AT and the uncured group BT versus AT. (E) Relative abundances of nine *Prevotella* lineages presented as bar plots. *P* values were calculated with the Wilcox test to assess the significance of the relative abundance in response to metronidazole between the BT and AT groups. Each bar shows the mean value, and error bars show 95% confidence intervals.

We used the same method to observe more intraspecies variations in *Prevotella* in the vagina. These genetic variations were also widely distributed in reference genes and present as 250 unique *Prevotella* 16S rRNA gene OTUs (Fig. 4A and Table S2). These 16S rRNA gene sequences have at least 1% genetic differences, and most occurred in V4 with 38 SNPs (Fig. 4B). Full-length 16S rRNA gene data were obtained from 196 mutants in *Prevotella*, while only 106 and 111 mutants were observed in V1 to V3 and V3 to V5, respectively (Fig. 4C). The different *Prevotella* species were identified by the full 16S rRNA gene sequences on the different levels.

Our results further confirm the abundance of *Prevotella* in the female vaginal microbiome and further supplement the 16S rRNA gene database. Moreover, a total of 15 *Prevotella* species were identified in the vagina and distributed in 11 phylogenetic lineages, among which *P. timonensis* was separated into two lineages (Fig. 4A). The substantial genetic diversity and numerous intraspecies variations were revealed by comparative analysis of 16S rRNA gene. In addition to the annotated *Prevotella* sequences, most novel *Prevotella* sequences were from *P. bivia* (*P. bivia*, P. amnii, and *Prevotella* sp. oral clone DA058), *P. timonensis*, *P. amnii*, P. disiens, *P. corporis*, P. buccalis, P. bergensis (including *P. bergensis* and *Prevotella* sp. *strain* S7-1-8), and *P. bivia*; our data supplemented sequences of these species by 2,400%, 2,100%, 750%, 625%, 350%, 190%, 133%, and 90%, respectively, a new sequence *P. timonensis* (*P. timonensis* and *Prevotella sp.* strain BV3P1) which added new reference data for the present database (Fig. 4A).

The distribution of mutants contained in *Prevotella* species was different in the cured group (BT versus AT) and uncured group (BT versus AT) (Fig. 4D). Before and after treatment shared 7 strains in the cured group but had 113 strains specific before treatment and 1 strain specific after treatment. There were 53 shared mutants, with 7 specific mutants before treatment and 6 specific mutants after treatment in the uncured group (Fig. 4D).

We also examined the type and abundance of *Prevotella* species before and after treatment. Decreasing abundances of *Prevotella* species were observed in the cured and uncured groups (Fig. 4E). *P. amnii*, *P. timonensis*, and *P. bivia* complex (*P. bivia*, *P. amnii*, *and Prevotella* sp. oral clone DA058) show significant differences before and after treatment (*P < *0.05) compared to other *Prevotella* species (Fig. 4E and Fig. S6A).

Compared to the reference literature, we also noticed that 7 of 17 species of *Prevotella* have not been previously reported in the vagina and were thought to be only in the oral cavity, gut, and skin (Table S3). The remaining species have not been reported or deemed as associating with BV. These novel *Prevotella* species could help us better understand the vaginal microbiota network in BV patients.

### Distinct strain-level associations in both cured and uncured patients.

**FIG 5:**
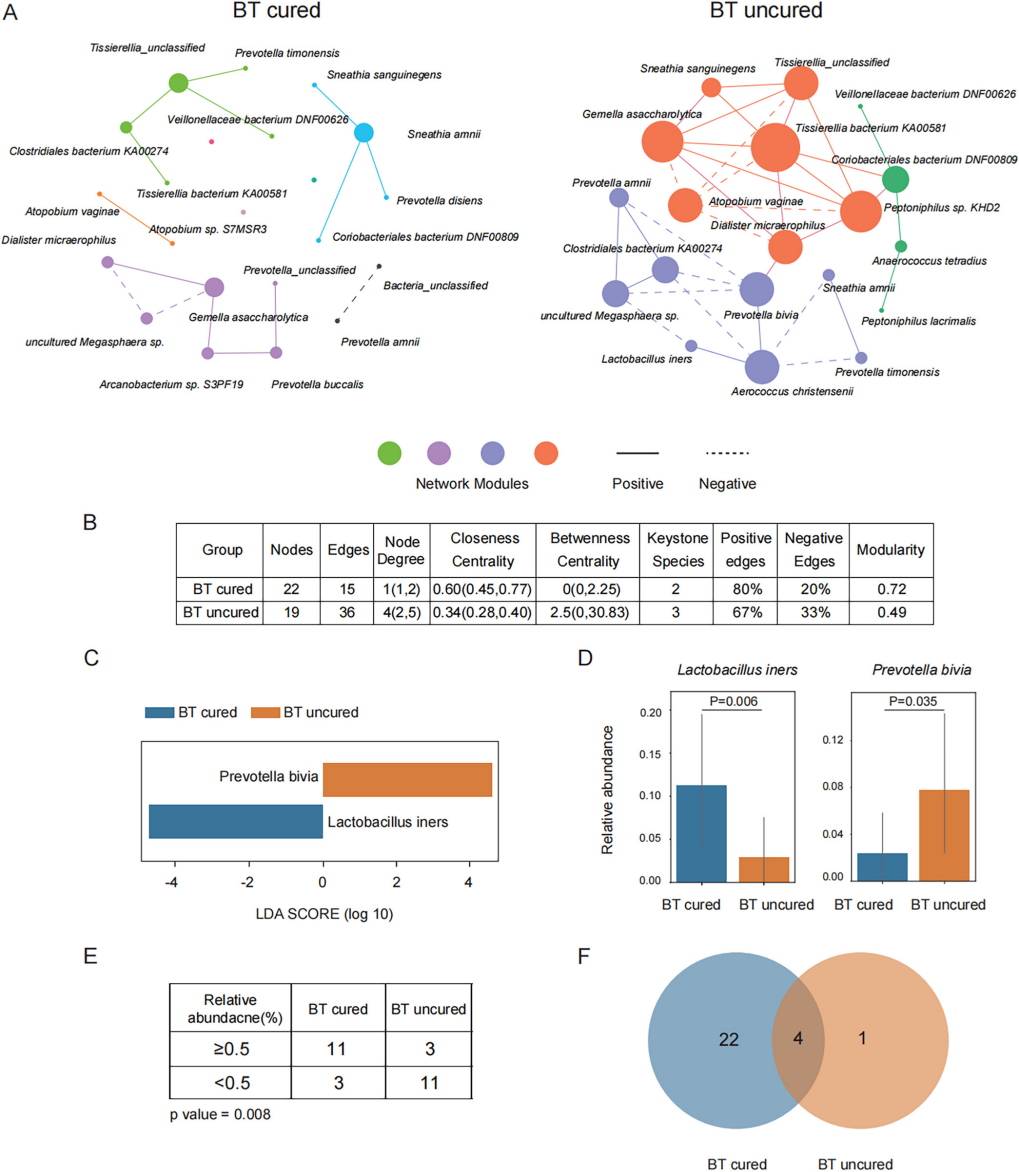
Identification of differences in the microbiota of cured and uncured patients before metronidazole treatment. (A) Microbiota cooccurrence networks in cured and uncured patients before metronidazole treatment. Node sizes are shown, proportional to node degree values, where node degree is defined as the number of edges connected to the node. Nodes in networks are color-coded to reflect the different bacterial modules to which they belong. Edges reflect a significant Spearman correlation (*P* ≤ 0.05, dashed line; *R* < –0.4, solid line; *R* > 0.4). Keystone species refer to the species in each network when values of node degree, closeness centrality, and betweenness centrality exceeded the 75th percentile of each of those parameters. (B). Comparison of microbiota network parameters in cured and uncured patients. (C) Significantly differentially distributed genera (LDA score, >4) between cured and uncured patients before metronidazole treatment. (D) Different relative abundances of *L. iners* and *P. bivia.* Each bar shows the mean value, and error bars show 95% confidence intervals. (E) Cutoff of *L. iners* abundances to predict the clinical outcome (Chi-square test, *P* < 0.05). (F) Distribution of mutants of *L. iners* in cured and uncured patients before metronidazole treatment.

Cooccurrence networks were constructed and used to examine interbacterial correlations in the two clinical groups (12). In an attempt to systematically correct inconsistencies in the official taxonomy of bacteria, due to short read lengths, we constructed the cooccurrence network diagram among bacteria to explore the relationships among strains and inferred groups (Fig. 5A). Compared with the cured group, the structure of the core network in the uncured group appeared to be tightly clustered around several pathogens, including T. bacterium KA00581, *P. bivia*, and Dialister micraerophilus, indicating a strong correlation between microbiomes within their hosts (Fig. 5A). Network connectivity and network modularity were also evaluated by searching for optimal modularity in graph partitioning. We identified four and three modules in the cured and uncured groups, respectively; however, there were clearly more edges and connections among microbial communities in the uncured group, suggesting a complex microbial network (Fig. 5A). In addition, we quantified a series of network parameters and found differences in closeness centrality, keystone species (key nodes in the network defined by Gephi), and positive/negative edges, in addition to modularity (Fig. 5B). Since large differences have been observed between cured patients and uncured patients, these data also suggested that these networks may be potential biomarkers for the prediction of clinical outcomes.

Through further investigation of differences in *Lactobacillus* and *Prevotella* species before treatment between the cured and uncured groups by LEfSe, we found that a high abundance of *L. iners* before treatment is a marker of curative clinical outcomes and that a high abundance of *P. bivia* before treatment is a marker of noncurative clinical outcomes (Fig. 5C). These data provide potential markers which may help to distinguish patients likely to respond positively to metronidazole treatment (Fig. 5D). Since higher abundances of *P. bivia* have been observed in uncured patients before the treatment than in cured patients, the lower abundance *P. bivia* may be a potential biomarker before the treatment. However, *P. bivia* located in two phylogenetic lineages (Fig. 4A); one of them displayed a significant decrease after treatment, whereases the other did not (Fig. 4E). This result showed that metronidazole was ineffective for at least for some *P. bivia*. As a large amount of *P. bivia* was detected in the vaginal microbiome of BV patients, clinicians should choose antibiotics to which *P. bivia* is sensitive to treat BV and improve the cure rate of BV (27).

Additional investigations suggested that patients with less than 0.05% *L. iners* were at a significantly increased risk of uncured outcome by metronidazole treatment (Chi-square test, *P* < 0.05). The area under the concentration-time curve (AUC) value is 0.8 below the 0.05% level (Fig. 5E). We also compared the mutants of *L. iners* before metronidazole treatment in the cured and uncured groups. There were 4 shared mutants in the 2 groups, 22 specific mutants pretreatment and 1 specific mutant pretreatment (Fig. 5F). These data suggested that different mutants of *L. iners* displayed various levels of protection during metronidazole treatment, and several specific mutants of *L. iners* might be a benefit to the metronidazole treatment.

## DISCUSSION

**FIG 6:**
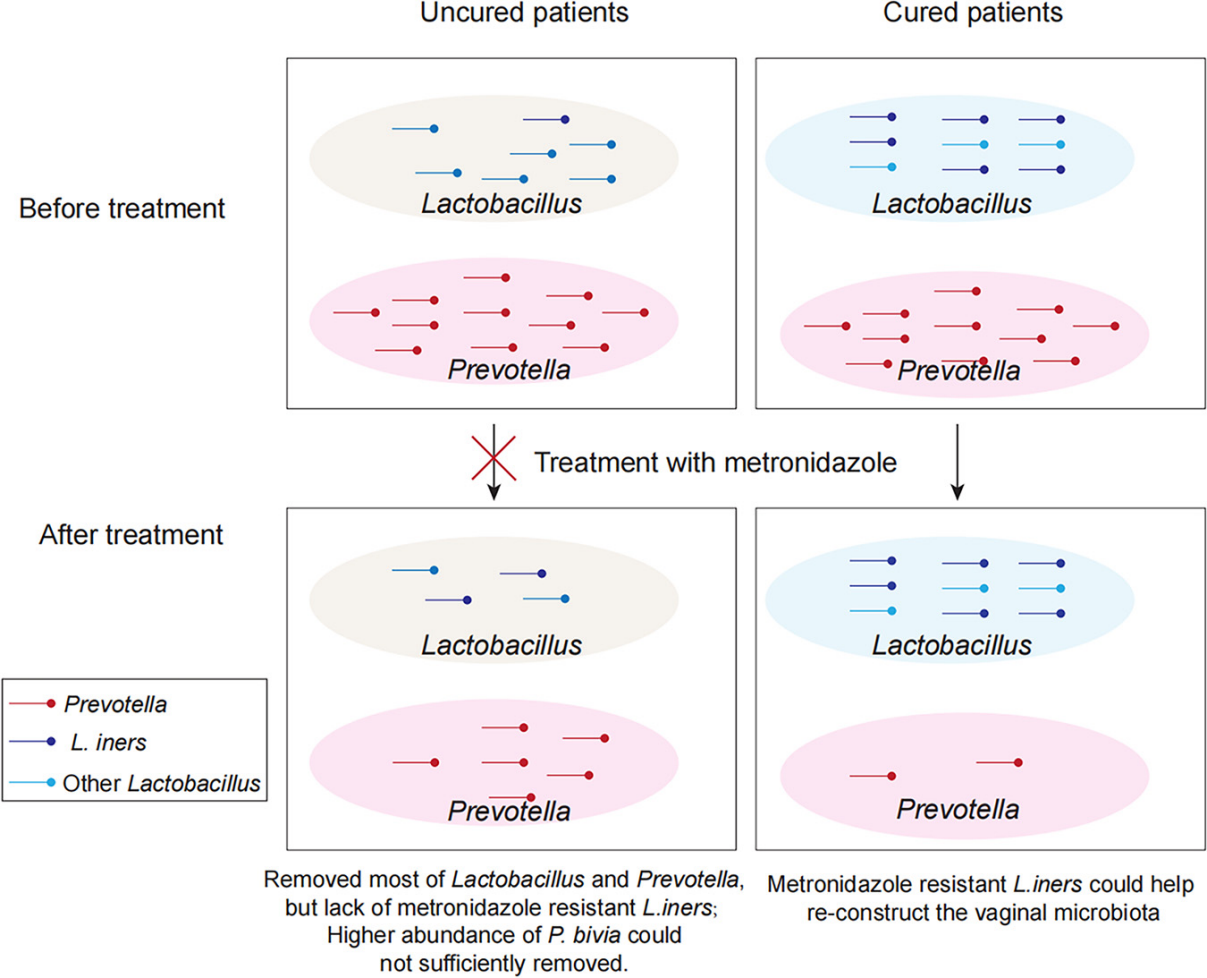
Framework of clinical outcomes of BV from the metronidazole treatment. The treatment of metronidazole reduced frequencies of both *Lactobacillus* and *Prevotella*. However, the cured outcome of BV is highly associated with the abundance of *Lactobacillus*, but not *Prevotella*.

BV has a high recurrence rate of approximately 80% 3 months after effective treatment with metronidazole or clindamycin (28). A number of previous studies have demonstrated significant alterations in the vaginal microbial composition in women with BV after antibiotic treatment (29, 30). Unfortunately, there are limited reports of biomarkers that can predict the outcomes of BV treatment. Therefore, we used 16S rRNA gene full-length sequencing, increased the discrimination of microbiota, explored various responses of bacteria in the vagina, and raised 3 predictors (network of microbiota, *L. iners*, and *P. bivia*) for clinical outcomes of BV treatment (Fig. 6).

Our observations of novel bacteria belonging to the genus *Lactobacillus* or *Prevotella* illustrate the important roles of key bacteria in the disease BV. *Prevotella* includes more than 50 species distributed in different natural habitats and body sites (31). Adopting the full-length 16S rRNA gene sequences, we identified *Prevotella* as a diverse genus and found 17 species of *Prevotella* in the vagina of Chinese women. Among these species, 7 novel *Prevotella* species had not been identified previously in the human vagina or even in animals as previously reported (Table S2). Previous research indicated that, in the vaginal microbiome, the most common *Prevotella* species related to BV were *P. bivia*, *P. timonensis*, *P. buccalis*, *P. disiens*, and *P. corporis* (31), which are consistent with our results. In addition, we observed that metronidazole treatment can significantly inhibit the relative abundance of *Prevotella* at the genus level in the cured group and the uncured group. Metronidazole resistance has been reported in the clinic (32), more specifically, for P. buccae (33) and *P. bivi*a (27). Whether all *Prevotella* species are sensitive to metronidazole requires more studies to be confirmed. In addition, *P. bivia* often cooccurs with Gardnerella vaginalis (34). *Gardnerella* spp. were observed in 95% to 100% of BV patients (35). However, *Gardnerella* spp. often form a constituent of the vaginal microbiota of healthy, asymptomatic women of all ages (16, 36). One possibility is that only certain species of *Gardnerella* are pathogenic and others are natural commensals. Therefore, FLAST, which improves discrimination of the vaginal microbiota, aids in the study of key species that affect treatment outcomes.

The healthy vaginal microbiota is dominated by *Lactobacillus* (37), which most frequently consisted of L. crispatus, L. jensenii, L. gasseri, and *L. iners* (38). We used FLAST sequencing to identify additional *Lactobacillus* species that had not previously been detected and were very widely distributed. The role of *L. iners* in the treatment of BV has not been widely reported. Adopting FLAST, we found that different mutants of *L. iners* presented significant changes in the cured and uncured groups before treatment. The treatment of BV with metronidazole, targeting these nonoptimal bacteria, is followed by the expansion of *L. iners* (39). The BV patients with a high level of *L. iners* before treatment were more likely to be cured. However, *L. iners* communities are less stable than those dominated by other *Lactobacillus* species (40, 41), and this increases the likelihood that women transition back to BV (42). However, the initial abundance of *L. iners* might be associated with the effect of metronidazole treatment. *L. iners* may have clonal variants that in some cases promote a healthy vagina and in other cases are associated with dysbiosis and disease (40).

FLAST is a powerful tool to identify novel bacteria from full-length 16S rRNA gene sequences, which is of great significance for research into the vaginal microbiome. To avoid potential sequencing errors, only sequences detected in multiple samples with high sequencing read frequencies were included. Furthermore, short16S rRNA gene sequences were removed, and closely related sequences (similarity, >99%) were combined for further investigation. By applying these strict thresholds, we confidently identified a large number of bacteria that persist in the human vagina. Compared to whole-metagenome sequencing, the metagenomic sequencing strategy on single molecular information of full length 16S rRNA gene provides clearer information to determine observed species. Also, the novel full-length 16S rRNA gene sequences would benefit to the global comparison of microbiomes, which could overcome the limitation of sequencing strategies on different genes used in various surveillances (43).

**Conclusion.** The 16S rRNA gene full-length sequencing method provided us with a better understanding of the changes in the microbiome of BV before and after metronidazole treatment. Three biomarkers were found to predict treatment outcomes, network of microbiota, *L. iners*, and *P. bivia*. Except for *P. bivia*, most *Prevotella* species are sensitive to metronidazole treatment. In addition, for BV patients in whom *L. iners* is dominant, simple bacterial connections or a low abundance of *P. bivia* in the vaginal microbiome is easy to cure with metronidazole treatment. Although it is not clear whether *L. iners* is beneficial or harmful, we speculate that it may be insufficient to maintain the vaginal microbiota. Thus, supplementary therapy with optimal species or targeted treatment of these nonoptimal bacteria may help to reduce BV recurrence and improve the cure rate. Novel treatment approaches are urgently needed to address this critical health care challenge.

## MATERIALS AND METHODS

### Study population and design.

From March 2017 to December 2019, we conducted a case-control study to explore the potential biomarkers for the outcome of metronidazole treatment. This study was approved by the ethics committee of Peking University First Hospital in Beijing, China, and conducted in accordance with the Declaration of Helsinki.

Premenopausal women 18 to 50 years of age were recruited to be screened after providing written informed consent. Participants were excluded if they met any of the following criteria: menstruation, pregnancy, allergy to metronidazole, and long-term use of immune-suppressants. Within 72 h prior to samples being taken, the women had no sexual intercourse or any products for vaginal use. No antibiotics were used in the month prior to enrollment. Those who had other systematic diseases were also excluded. The enrolled patients could not have other genital tract infections, such as Chlamydia trachomatis, gonococcal vaginitis, vulvovaginal candidiasis, Trichomonas vaginalis, and human papilloma virus (HPV) infection. A cervical swab was obtained for nucleic acid testing for gonorrhea, C. trachomatis, and HPV. Only BV patients were recruited as determined by microscopic examination.

Eligible woman with BV had to meet at least three of four Amsel criteria (44) and have a Nugent score of 7 to 10 (45) by Gram stain (visit 1). These BV patients were treated with a standard 5-day metronidazole course (0.75% metronidazole vaginal gel from NiMeiXin, Tongfang Pharmaceutical Group Co. Ltd.; 5 g once daily) (10). The conclusion of BV treatment was classified as uncured (Nugent score, 7 to 10), intermediate BV (Nugent score, 4 to 6), or healthy (Nugent score, 0 to 3) (visit 2) (11). Clinical cure was defined as resolution of three of four Amsel criteria. Bacteriologic cure was defined as a Nugent score lower than 7, and therapeutic cure as both a clinical cure and bacteriologic cure (46).

The uncured patients were given metronidazole treatment prolonged to 10 days or were switched to clindamycin for another 5 to 7 days (clindamycin phosphate vaginal gel from Zhejiang Xianju Pharmaceutical Co., Ltd.; 5 g once daily). We asked the uncured patients return to the clinic within 1 to 2 weeks after complementary therapy to provide a vaginal discharge sample for microecological examination (visit 3). The cured patients were asked to return to the clinic within 3 to weeks after treatment (visit 3).

Biological samples were collected before treatment (visit 1), between 7 and 10 days following the conclusion of treatment (visit 2), and again 28 to 32 days posttreatment (visit 3). At visits 1 and 2, two vaginal swabs were obtained from the standard anatomical site (lateral vaginal wall) (47). One of vaginal swab was sent to the microbiology laboratory of the First Hospital of Peking University for Gram’s stain. The other vaginal swab was placed in 1 mL of phosphate-buffered saline (PBS) and stored at −80°C in the laboratory, and the fluid used for genomic DNA extraction (14, 48). These genomic DNAs were applied to detect the vaginal microbiota using FLAST.

At visits 1 and 2, we collected 92 vaginal samples from 46 patients before and after metronidazole treatment for FLAST sequencing analysis. We excluded 27 unqualified DNA samples and obtained 168.1 Gb of data from 65 vaginal samples, covering 56 paired vaginal samples from 28 patients and 9 unpaired samples from 9 patients (Fig. S7). According to the unique tag encoded in both flanks, we obtained 476,780 unique 16S rRNA gene sequences (>1,200 bp). The sequencing depths of full-length 16S rRNA gene sequences ranged from 2× to 100×. At visit 3, only one sample of vaginal secretions needed to be collected for microscopic examination to assess the long-term outcome of BV treatment.

### Library construction for FLAST.

The basic theory and protocols of FLAST are similar to those described in the previous report on the 16S rRNA gene full-length single-molecular library construction (49). To increase the stability and performance, we updated the protocol with customized kits and steps. Briefly, bacterial DNA was first extracted using a fecal DNA extraction kit (Qiagen, Hilden, Germany). The concentrations of DNA were quantitative and qualitatively measured, and we removed samples with a concentration of <1 ng/μL to ensure high-quality samples (Qubit 4.0, Thermo Fisher Scientific, USA). The major steps for library construction included adaptor ligation, amplification, read-tag library construction, and linked-tag library construction. (i) Adaptor ligation was performed by three cycles of full-length amplification with the primer (Table S4, primer set 1). (ii) Amplifications were conducted under the reaction system containing 10 ng DNA, 25 μL Platinum SuperFi PCR master mix (Thermo Fisher Scientific), primers (Table S4, primer set 2), and nuclease-free water for a total volume of 50 μL. PCR was used for the initial denaturation at 95°C for 5 min, 3 cycles of denaturation at 95°C for 30 s, annealing at 58°C for 30 s, elongation at 72°C for 1 min, and finishing with a final extension at 72°C for 5 min. These products were purified using 0.7× Hieff NGS DNA selection beads (Yeasen, China) and eluted in 20 μL nuclease-free water. (iii) The read-tag library was constructed with a customized protocol using a Nextera library preparation kit (Illumina, USA). The reaction was prepared with 10 ng product, 5 μL tagmentation DNA buffer (Illumina), 0.5 μL tagmentation DNA enzyme (Illumina), and 20 μL nuclease-free water. After incubation at 55°C for 5 min, the tagmentation products were amplified with the reaction system of 25 μL Platinum SuperFi PCR master mix (Thermo Fisher Scientific, USA), 1 μL 10 μM primers (Table S3, primer set 3), and 12 μL nuclease-free water. The PCR procedure comprised initial denaturation at 95°C for 5 min, 8 cycles of denaturation at 95°C for 30 s, annealing at 58°C for 30 s, elongation at 72°C for 1 min, and finishing with final extension at 72°C for 5 min. The product was purified using 0.7× Hieff NGS DNA selection beads (Yeasen, China). (iv) The linked-tag library was constructed with a circularized in an intramolecular blunt-end ligation reaction and two rounds of PCRs. The ligation reaction system contained 1 μL T4 ligase (Thermo Fisher Scientific), 2 μL 10× T4 ligase buffer (Thermo Fisher Scientific), and nuclease-free water. The reaction was incubated at 16°C for 60 min. The following two rounds of PCR were conducted with the same reaction system and PCR program as described above, but with different PCR primers (Table S4, primer sets 4 and 5, respectively). After quality control, paired libraries (concentration of read-tag library, >5ng/μL; linked-tag library, >1 ng/μL) were adopted for sequencing with a NovaSeq 6000 instrument (Illumina).

### Bioinformatics analysis for FLAST.

To obtain taxonomic information in each sample, the bioinformatic analysis pipeline was developed. Adaptor ligation was performed with three cycles of full-length amplification with the primer (Table S1, primer set 1). During the synthesis, we used the unique tag to tag the individual 16S rRNA gene molecules on both termini. First, unique tag pairing relationships were found from the linked-tag library with Cutadapt v1.2.1 (50). Then, all sequences corresponding to each pair of unique tags from the read-tag library were assembled to generate a full-length 16S rRNA gene sequence using the software SPAdes v3.13.1 (51) with default parameters. After we obtained and qualified full-length 16S rRNA gene sequences (length, ≥1,200 bp) from all of the samples, these sequences were clustered into operational taxonomic units (OTUs) with 99% sequence similarity using VSEARCH in QIIME 2 (52, 53). These OTUs were annotated according to reference database SILVA_132_SSURef_Nr99 (54) using mothur v1.42.0 (55).

The compositional diversity of each sample was analyzed using the R v3.6.1 package (vegan v2.5-3). The compositional comparison among groups was conducted using LEfSe v 1.0 (56), in which the significantly different distributed species or genera were identified with linear discriminant analysis (LDA) analysis if the LDA score was >4.

The cooccurrence network was constructed using Gephi v0.9.2 (57). The edges of the network were defined by the correlations of each pair of bacteria, which were calculated with the Spearman method in SparCC (58). To simplify the network, only the edges with a correlation of >0.4 and *P* value ≤ 0.05 were retained in network.

To explore the intradiversity in *Lactobacillus* and *Prevotella*, we first aligned all sequences observed in this study, as well as OTU sequences in the SILVA_132_SSURef_Nr99 database (only sequences of >1,200 bp with undetermined bases [N] of <1% were retained). Clustal W v2.0.10 was used for multiple sequence alignment (59). Customized Perl scripts were developed to calculate the distribution of bases in each position. The potential sequencing errors were removed if N was over 10%. The major allele base in each position was considered and connected as a consensus sequence. Mutations of each sequence were obtained by comparison to the reference sequences if the bases with the highest proportion in a given position were different from those of the consensus sequences.

To illustrate the relationships among species, we drew two kinds of heatmaps (heatmap of species abundance matrix and heatmap of SNP presence/absence matrix). Both heatmaps were drawn using the R package (pheatmap v1.0.12). Lineages were produced with cutree_col using pheatmap on the SNP presence/absence matrix.

### Statistical analyses.

*P* values were calculated by Wilcoxon rank-sum test to assess the significance of age between cured and uncured groups, Nugent score and pH value of vaginal discharge between the cured and uncured groups before metronidazole treatment, and the between cured and uncured groups after metronidazole treatment. Permutational multivariate analysis of variance (PERMANOVA) with the Bray-Curtis distance was employed to compare the community structure between BT and AT in the cured group, BT and AT in the uncured group, and the cured and uncured groups before metronidazole treatment. Correlations were also evaluated by Spearman correlation between these groups. *P* values were calculated by Wilcoxon signed-rank test to assess the significance of relative abundance in response to metronidazole between BT and AT in the cured group, BT and AT in the uncured group, and the cured and uncured groups before metronidazole treatment. In order to assess the abundance of *L. iners* used to distinguish treatment outcome, the *P* value of the Chi-square test and the AUC were calculated between the uncured and cured groups before metronidazole treatment. The Wilcoxon rank-sum test to was used to calculate the *P* value of metabolic pathway changes in BT versus AT in the cured group, BT versus AT in the uncured group, and BT versus BT in both groups.

### Ethics statement.

The studies involving human participants were reviewed and approved by The Ethics Committee of Peking University First Hospital (V2.0/201504.20), and written informed consent was obtained from all participants. The patients/participants provided their written informed consent to participate in this study.

### Data availability.

The data sets analyzed for this study can be found in the National Genomics Data Center (https://ngdc.cncb.ac.cn/) under accession number CRA004911.
