# Supplementary material for: Single-Molecule Approach to 16S rRNA for Vaginal Microbiome Signatures in Response to Metronidazole Treatment
Source: Microbiol Spectr. 2023 May 18;11(3):e01706-22. doi: 10.1128/spectrum.01706-22 (PMC10269914; doi:10.1128/spectrum.01706-22)
Supplement: Supplemental file 1 — Supplemental material. Download spectrum.01706-22-s0001.pdf, PDF file, 1.2 MB [file spectrum.01706-22-s0001.pdf]

Supplemental Materials

Supplemental Figures

Supplemental Figure 1. Comparison of microbiota between cured and uncured patients before and after metronidazole treatment

(A-**C**) PCoA analyses were performed at phylum level. (A) AT vs. BT in uncured group, (B) Cured group vs. uncured group before the treatment, and (C) AT vs. BT in the cured group.

(D-**F**) PCoA analyses were performed at genus level. (D) AT vs. BT in uncured group, (E) Cured group vs. uncured group before the treatment, and (F) AT vs. BT in the cured group.

Sample distance was measured using the non-phylogenic Bray-Curtis distance method. The overlap zones represent the range of two-component values with the 95% confidence intervals of observed data for each outcome group. Correlations between groups were also computed by Spearman correlation. Statistical significance was measured using Permutational ANOVA (PERMANOVA). (BT, before treatment; AT, after treatment)

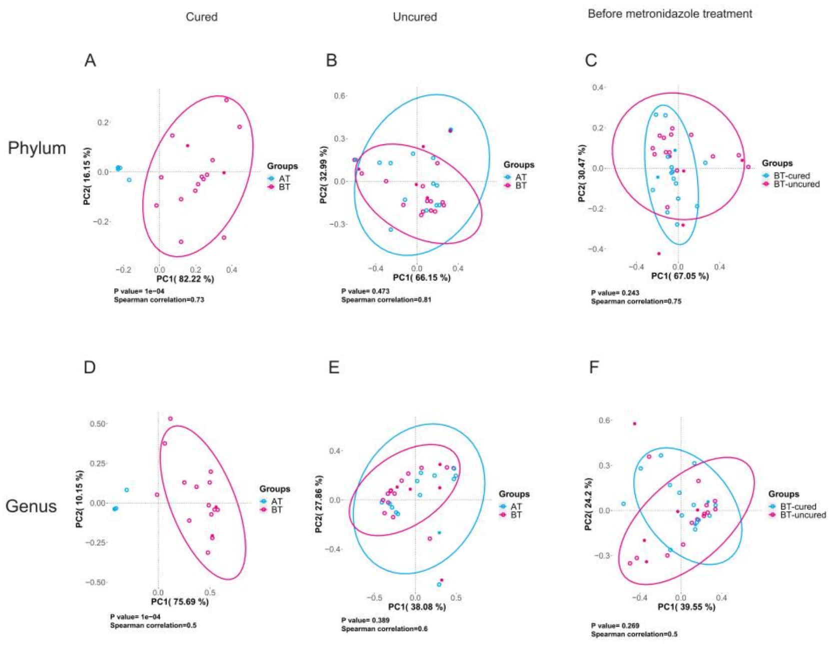

Supplemental Figure 2. Comparison of sequences used in this study (27F primer) and the reference sequence of other vaginal bacteria (*Gardnerella vaginalis*: MH898664.1; *Lactobacillus*: AEXK01000007; *Atopobium* : ACGK02000001; *Prevotella*: AB547673; *Megasphaera*: FMFF01000017)

27F primer	A G A G T T T G A T C M G G C T G A G
Gardnerella	A G G G T T C G A T T C G G C T G A G
Lactobacillus	A G A G T T T G A T C C G G C T C A G
Atopobium	A G A G T T C G A T C C G G C T C A G
Prevotella	A G A G T T T G A T C C G G C T C A G
Megasphaera	A G A G T T T G A T C C G G C T C A G

1492R primer	A A G T C G T A A C A A G G T A R C Y
Gardnerella	A A G T C G T A A C A A G G T A G C C
Lactobacillus	A A G T C G T A A C A A G G T A G C C
Atopobium	A A G T C G T A A C A A G G T A G C C
Prevotella	A A G T C G T A A C A A G G T A A C C
Megasphaera	A A G T C G T A A C A A G G T A G C C

Supplemental Figure 3. Taxonomic diversity of cured and uncured patient samples at species-level

(A) Heat maps of abundance and prevalence of core microbiota of the top 15 species in cured and uncured patient samples pre- and post treatment.

(B) Significantly differentially distributed genera (LDA score >4) in cured and uncured patient samples pre- and posttreatment. (blue: before treatment; pink: after treatment)

(C) Relative abundances of dominant species pre- and post treatment among cured and uncured group. Bar plot shows the mean value and error bars shows 95% confidence intervals. Different colors represent different treatment stages (bule and green: before treatment; orange and red: after treatment) and outcomes (blue and orange: cured; green and red: uncured).

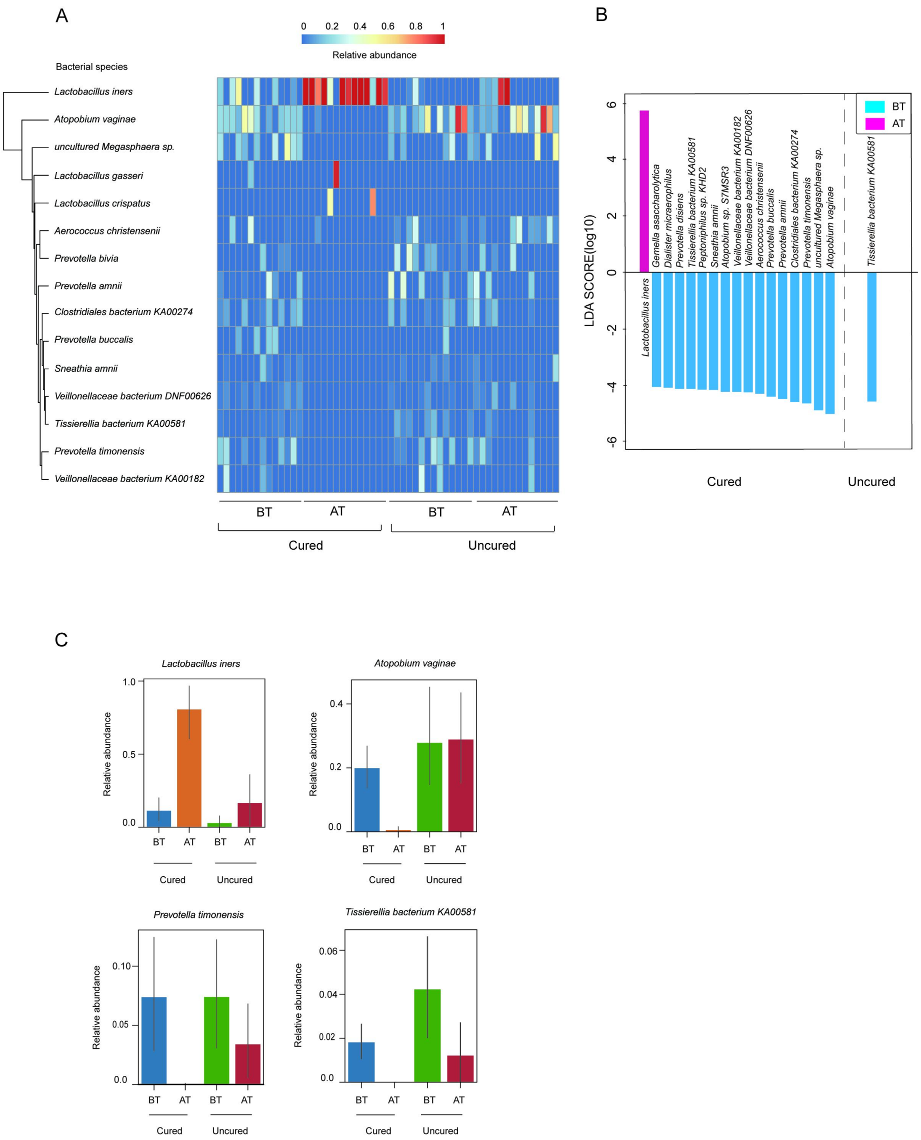

Supplemental Figure 4. Comparison of microbiota metabolic pathways in human vagina cured and uncured patients before and after metronidazole treatment

Relative abundance of identified MetaCyc in cured and uncured patient samples pre- and posttreatment. The functional groups accounted for at least 1% and were among the top 5 relative richness changes before and after treatment. P-values were calculated by Wilcoxon rank-sum test to assess the significance. Box limits indicate the range of the central 50% of the data, with a central line indicating the median value. Lines extend from each box to capture the range of the remaining data, with dots placed past the line edges to indicate outliers. Different colors represent different treatment stages (blue: before treatment; orange: after treatment).

(A) AT vs. BT in the cured group: p-value < 0.05;

(B) AT vs. BT in uncured group: p-value = 0.022;

(C) Cured vs. Uncured group BT: p-value < 0.05.

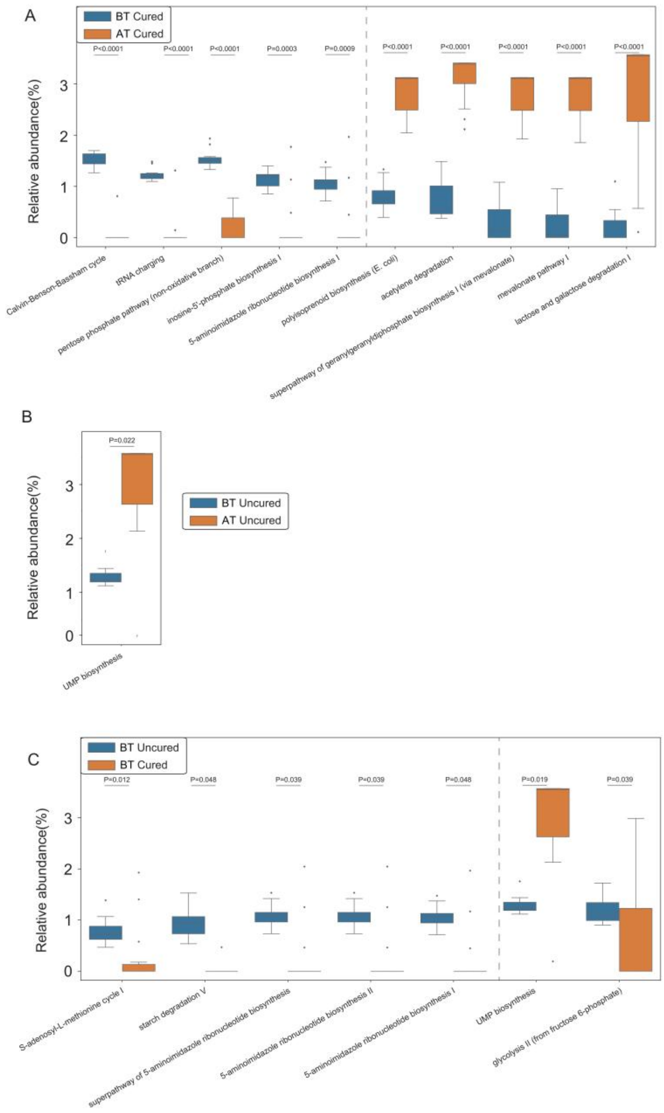

Supplemental Figure 5. The *Lactobacillus spp.* of abundance comparing and unrooted phylogenetic tree.

(A) The linear relations of the abundance (log10) of different *Lactobacillus* spp. Observed in the different groups (before treatment in cured, after treatment in cured, before treatment in uncured, after treatment in uncured). The linear regression model fits by Seaborn. Lmplot Version0.11.1. Each point represented individual sample, and the color corresponded to their group.

(B) Unrooted phylogenetic tree of the genus of *Lactobacillus.* Unrooted phylogenetic tree of *Lactobacillus* genomes from previous reference bacterial isolates and present study. The out circle indicated the species that annotated in the reference database, and the inner circle indicated the source of genome data.

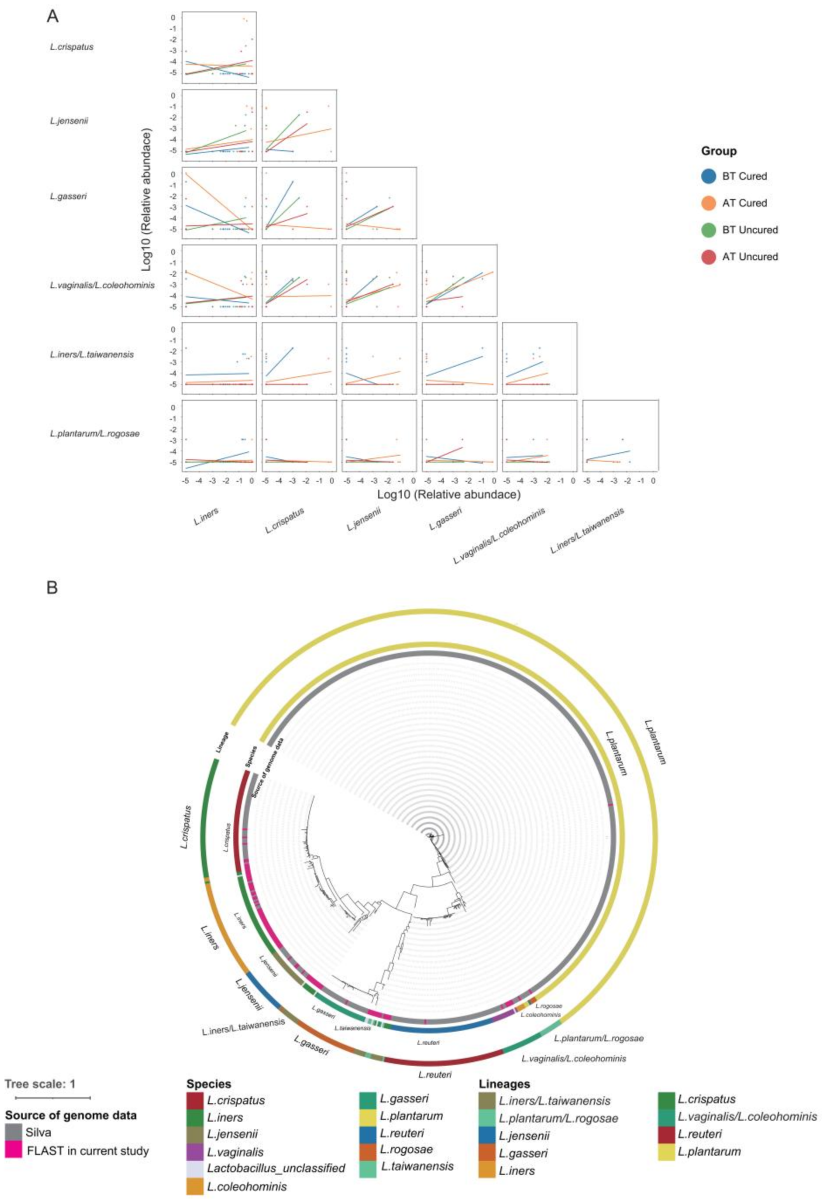

Supplemental Figure 6. The *Prevotella spp.* of abundance comparing and unrooted phylogenetic tree.

(A) The comparison of abundance of *Prevotella* in uncured group (AT vs. BT). P-values were calculated by Wilcox-test.

(B) Unrooted phylogenetic tree of the genus of *Prevotella*. Unrooted phylogenetic tree of *Prevotella* genomes from previous reference bacterial isolates and present study. The out circle indicated the species that annotated in the reference database, and the inner circle indicated the source of genome data.

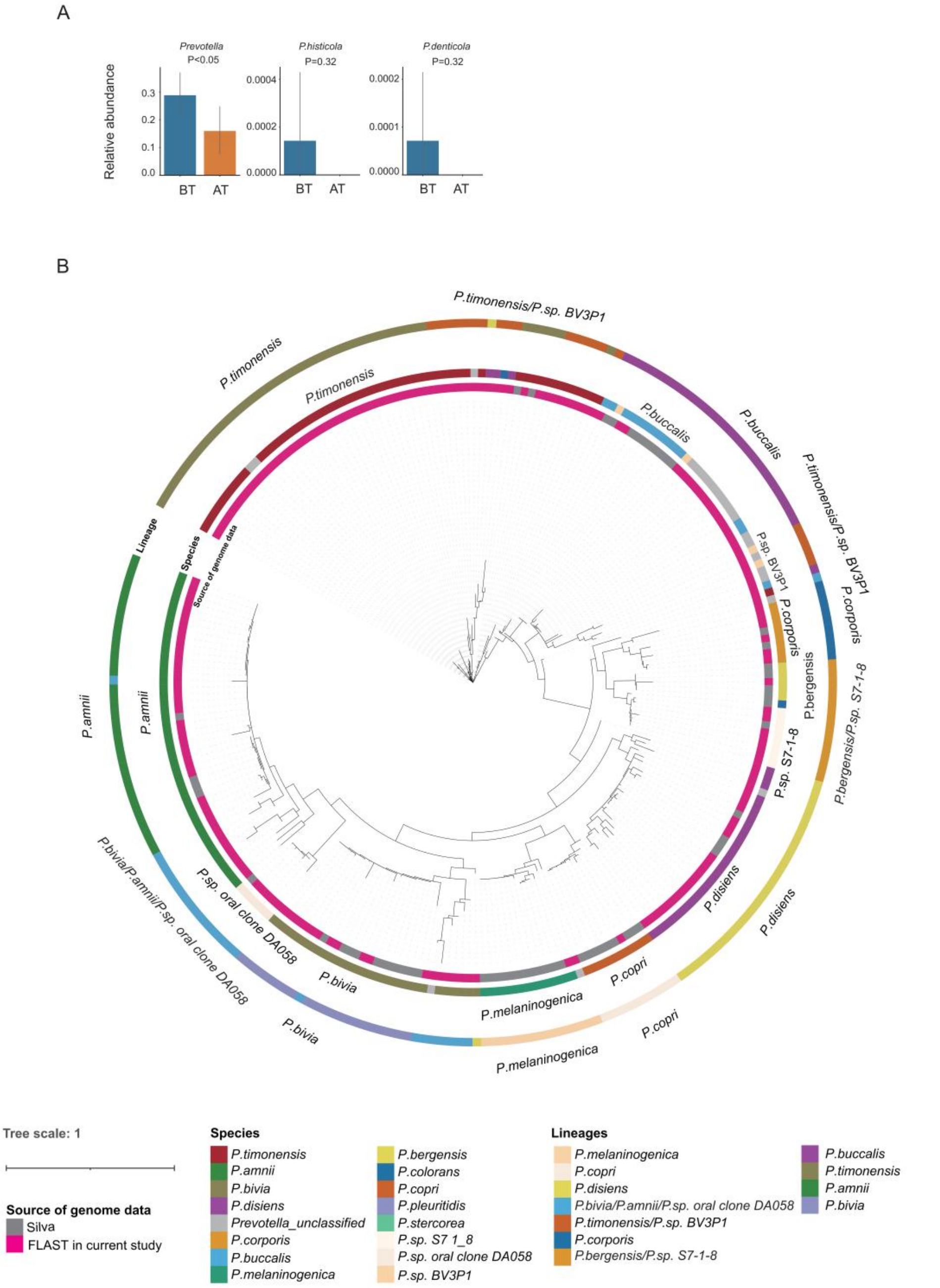

Supplemental Figure 7. The process of taking samples.

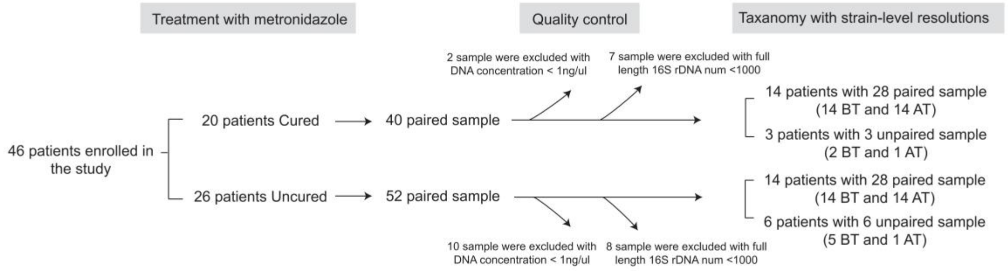

Supplemental Tables

Supplemental Table 1. Treatment Outcomes of BV patients.

**Clinical ID**	**Visit 2** **Treatment Outcome**	**Visit 3** **With Additional Treatment**	**Clinical ID**	**Visit 2** **Treatment Outcome**	**Visit 3** **Without Additional Treatment**
B-5	Uncured	Health	**Patient1**	Cured	BV
**Patient29**	Uncured	Health	**Patient2**	Cured	Health
**Patient30**	Uncured	BV	B-121	Cured	Health
**Patient31**	Uncured	BV	**Patient4**	Cured	BV
B-37	Uncured	Health	**Patient6**	Cured	BV
**Patient33**	Uncured	BV	**Patient10**	Cured	Health
**Patient34**	Uncured	Health	**Patient13**	Cured	Health
B-71	Uncured	Health	**Patient14**	Cured	Health
B-79	Uncured	Health	B-162	Cured	Health
B-83	Uncured	BV	B-164	Cured	BV
**Patient37**	Uncured	Health	**Patient17**	Cured	Health
B-114	Uncured	BV	B-166	Cured	Health
**Patient5**	Uncured	Health	B-173	Cured	Health
**Patient7**	Uncured	Health	**Patient20**	Cured	Health
**Patient8**	Uncured	Health	**Patient21**	Cured	BV
B-141	Uncured	Health	**Patient22**	Cured	BV
**Patient11**	Uncured	Health	**Patient24**	Cured	Health
B-156	Uncured	Health	**Patient26**	Cured	Health
**Patient15**	Uncured	BV	**Patient28**	Cured	Health
B-161	Uncured	Health			
**Patient19**	Uncured	Health			
B-175	Uncured	Health			

**Patient23**	Uncured	BV			
**Patient25**	Uncured	BV			
B-193	Uncured	Cured			
B-202	Uncured	Cured			

* Diagnostic criteria for BV (Nugent score belonging to 7-10)and health (Nugent score belonging to 0-6) were described in the Methods section.

Supplemental Table 2. Mutant sites and regions of the new *Lactobacillus* and *Prevotella* species.

Reference **Supplementary data**.

Supplemental Table 3. Distribution characteristics of *Prevotella* species

***Prevotella* Species**	**Host sites**	**Previous reported in the vagina (Yes: Y, No: N)**	**Reference**
P. amnii	Vagina, amniotic fluid	Y	(Gottschick et al., 2017; Lawson et al., 2008)
P. bivia	Vagina	Y	(Randis and Ratner, 2019)
P. histicola	Vagina, oral cavity, gut	Y	(Downes et al., 2008; Marietta et al., 2016; Tett et al., 2021)
P. melaninogenica	Vagina, oral cavity, sputum	Y	(Andersson et al., 2013; Könönen and Gursoy, 2021)
P. denticola	Vagina, oral cavity	Y	(Kolenbrander et al., 2010; Tett et al., 2021)

P. sp. oral clone DA058	Oral cavity and gut	N	(Picchianti Diamanti et al., 2020; Siqueira and Rôças, 2013)
P. bergensis	Skin and soft-tissue abscesses	N	(Downes et al., 2006)
P. corporis	Vagina	Y	(Hillier et al., 1993)
P. copri	Gut and face	N	(Cani, 2018; Hayashi et al., 2007)
P. stercorea	Gut and face	N	(Hayashi et al., 2007; Hui et al., 2021)
P. pleuritidis	Pleural fluid	N	(Sakamoto et al., 2007)
P. timonensis	Vagina, breast abscess	Y	(Glazunova et al., 2007; van Teijlingen et al., 2020)
P. disiens	Vagina, gut, Bartholin abscess	Y	(Liss et al., 2019; Spiegel, 1991; Tett et al., 2021)
P. colorans	Wound	N	(Buhl et al., 2016)
P. sp. S7 1_8	Rumen and gut	N	(Dao et al., 2021; Nakayama et al., 2019)
P. buccalis	Vagina	Y	(Kumar et al., 2021)
P. sp. BV3P1	No report.	-	-

Supplemental Table 4. Primers for Library construction

Name	Primer
primer set 1: 27F	5'-CTCCACCCAGACTCATCCATNNNNNNNNNNNNNNGCGATCTAAGAGTTTGATCMTGGCTCAG-3'
primer set 1:1492R	5'-AGGGGGGCAAAGATGAAGATNNNNNNNNNNNNNNCGTACTAGTACGGYTACCTTGTTACGACTT-3'
primer set 2: F	5'-CTCCACCCAGACTCATCCAT-3'
primer set 2: R	5'-AGGGGGGCAAAGATGAAGAT-3'
primer set 3: F1	5'-GTGACTGGAGTTCAGACGTGTGCTCTTCCGATCTCTCCACCCAGACTCATCCAT-3'
primer set 3: F2	5'-GTGACTGGAGTTCAGACGTGTGCTCTTCCGATCTAGGGGGGCAAAGATGAAGAT-3'
primer set 3: R	5'-TCGTCGGCAGCGTCAG-3'
primer set 4: F	5'-GTGACTGGAGTTCAGACGTGTGCTCTTCCGATCTAAGTCGTAACAAGGTARCCGTACTAGTACG-3'
primer set 4: R	5'-TCTACACTCTTTCCCTACACGACGCTCTTCCGATCTCTGAGCCAKGATCAAACTCTTAGATCGC-3'
primer set 5: P7	5'-CAAGCAGAAGACGGCATACGAGATAGCAGGAAGTGACTGGAGTTCAGACGTG-3'
primer set 5: P5	5'-AATGATACGGCGACCACCGAGATCTACACAAACATCGACACTCTTTCCCTACACGAC-3'

* NNNNNNNNNNNNNN present as unique tag

Supplemental Table 5. Mutants distribution of *Lactobacillus*

Patients	OTUs identified in before treatment	OTUs identified in After Treatment	Shared number of OTUs	Group
**Patient1**	OTU0, OTU18, OTU19, OTU25, OTU28, OTU29, OTU31, OTU34, OTU36	OTU0, OTU18, OTU19, OTU20, OTU27, OTU28, OTU30, OTU31, OTU32, OTU33, OTU36, OTU38, OTU40, OTU43	6	Cured
**Patient2**	OTU0, OTU30, OTU35	OTU0, OTU14, OTU18, OTU19, OTU20, OTU29, OTU30, OTU32, OTU35, OTU36, OTU38, OTU39	3	
**Patient4**	OTU0, OTU2, OTU3, OTU7, OTU8, OTU9, OTU11, OTU14, OTU18, OTU20, OTU28, OTU30, OTU31, OTU32, OTU36, OTU39	OTU0, OTU2, OTU7, OTU11, OTU14, OTU15, OTU18, OTU19, OTU20, OTU30, OTU31, OTU32, OTU36, OTU37, OTU38, OTU42, OTU48, OTU50, OTU51	11	
**Patient6**	OTU0, OTU9, OTU12, OTU19, OTU20, OTU30, OTU32, OTU41, OTU49, OTU53, OTU54, OTU55, OTU56	OTU0, OTU4, OTU18, OTU19, OTU20, OTU32, OTU35, OTU36, OTU38, OTU39, OTU41, OTU42, OTU52, OTU53, OTU57	6	
**Patient10**	OTU0, OTU9, OTU41	OTU0, OTU1, OTU2, OTU4, OTU5, OTU6, OTU10, OTU13, OTU14, OTU18, OTU19, OTU20, OTU21, OTU27, OTU30, OTU32, OTU35, OTU36, OTU37, OTU38, OTU51, OTU62, OTU63, OTU64, OTU65, OTU66, OTU67, OTU68, OTU69, OTU70, OTU71, OTU72, OTU73, OTU75, OTU76, OTU77, OTU78, OTU79, OTU82	1	
**Patient13**	OTU1, OTU3, OTU4, OTU8, OTU9, OTU22, OTU23, OTU24, OTU85, OTU86, OTU87, OTU88, OTU89, OTU90, OTU91	OTU3, OTU4, OTU8, OTU9, OTU80, OTU85, OTU90, OTU92, OTU93, OTU94	6	
**Patient14**	OTU0, OTU19, OTU20, OTU26, OTU28, OTU33, OTU34, OTU36, OTU58, OTU61, OTU95, OTU96, OTU97, OTU98	OTU0, OTU18, OTU19, OTU20, OTU32, OTU33, OTU36, OTU61, OTU84	6	
**Patient17**	OTU0, OTU28, OTU38	OTU0, OTU2, OTU4, OTU14, OTU16, OTU19, OTU28, OTU30, OTU32, OTU47,	2	

		OTU59, OTU61, OTU81		
**Patient20**	OTU0	OTU0	1	
**Patient21**	OTU0, OTU8, OTU18, OTU46, OTU100, OTU101, OTU102, OTU103, OTU104, OTU105	OTU0, OTU9	1	
**Patient22**	OTU0, OTU17, OTU44	OTU0	1	
**Patient24**	OTU0	OTU0, OTU1, OTU5, OTU6, OTU14, OTU63, OTU64, OTU67, OTU74	1	
**Patient26**	OTU0, OTU26	OTU0, OTU19, OTU20, OTU28, OTU30, OTU39	1	
**Patient28**	OTU0	OTU0, OTU2, OTU14, OTU18, OTU19, OTU27, OTU28, OTU32, OTU99, OTU106	1	
**Patient5**			0	Uncured
**Patient7**	OTU0	OTU0, OTU28	1	
**Patient8**	OTU0	OTU0, OTU19	1	
**Patient11**	OTU0, OTU2	OTU0, OTU1, OTU4, OTU83	1	
**Patient15**	OTU0, OTU1, OTU2, OTU3, OTU4, OTU18, OTU28, OTU35, OTU37, OTU85	OTU0, OTU1, OTU2, OTU3, OTU4, OTU18, OTU19, OTU20, OTU29, OTU32, OTU36, OTU43, OTU45, OTU63, OTU83	6	
**Patient19**	OTU0	OTU0, OTU19, OTU27, OTU28, OTU32, OTU58, OTU61	1	
**Patient23**		OTU3, OTU60	0	
**Patient25**			0	
**Patient29**			0	
**Patient30**			0	
**Patient31**			0	
**Patient33**	OTU4	OTU4	1	
**Patient34**			0	
**Patient37**			0	

Supplemental Table 6. Mutants distribution of *Prevotella*

Patients	OTU found in Before Treatment	OTU found in After Treatment	Share number	Group
**Patient1**	OTU0, OTU1, OTU13, OTU21, OTU30, OTU32, OTU34, OTU48, OTU49, OTU50, OTU52, OTU54, OTU55, OTU56, OTU57, OTU58, OTU60		0	Cured
**Patient2**	OTU1, OTU6, OTU24, OTU25, OTU35, OTU49, OTU50, OTU55, OTU64, OTU65, OTU66, OTU67, OTU68, OTU69, OTU70, OTU71, OTU72, OTU73, OTU74, OTU82, OTU83, OTU84, OTU88		0	
**Patient4**	OTU0, OTU32	OTU1, OTU3, OTU44, OTU63, OTU85, OTU106, OTU108, OTU109	0	
**Patient6**	OTU2, OTU26, OTU29, OTU104, OTU124		0	
**Patient10**	OTU0, OTU1, OTU7, OTU13, OTU24, OTU28, OTU34, OTU49, OTU55, OTU78, OTU111, OTU146		0	
**Patient13**			0	
**Patient14**	OTU0, OTU2, OTU6, OTU7, OTU18, OTU20, OTU26, OTU29, OTU39, OTU54, OTU93, OTU94, OTU96, OTU97, OTU148, OTU149, OTU150, OTU151, OTU152		0	
**Patient17**	OTU1, OTU3, OTU6, OTU14, OTU16, OTU23, OTU24, OTU36, OTU49, OTU55, OTU63, OTU64, OTU67, OTU69, OTU70, OTU76, OTU77, OTU78, OTU81, OTU90, OTU105, OTU106, OTU107, OTU108, OTU109, OTU119, OTU127, OTU130, OTU155, OTU157, OTU158, OTU159, OTU160, OTU161, OTU162, OTU163, OTU164, OTU165, OTU166, OTU167		0	
**Patient20**	OTU0, OTU2, OTU8, OTU11, OTU15, OTU38, OTU39		0	

**Patient21**	OTU0, OTU2, OTU7, OTU8, OTU18, OTU20, OTU40, OTU96, OTU101, OTU169, OTU170, OTU171, OTU172, OTU173		0	
**Patient22**	OTU1, OTU3, OTU8, OTU9, OTU10, OTU12, OTU35, OTU41, OTU42, OTU175, OTU176, OTU177		0	
**Patient24**	OTU1, OTU3, OTU6, OTU63, OTU156		0	
**Patient26**	OTU1, OTU24, OTU49, OTU50, OTU52, OTU85, OTU180, OTU181		0	
**Patient28**	OTU0, OTU1, OTU5, OTU6, OTU9, OTU17, OTU54, OTU57, OTU64, OTU91, OTU122, OTU168, OTU182, OTU183, OTU184, OTU185		0	
**Patient5**	OTU0, OTU1, OTU9, OTU27, OTU28, OTU31, OTU32, OTU49, OTU52, OTU54, OTU62, OTU64, OTU111, OTU112, OTU113, OTU114, OTU115, OTU116, OTU117, OTU118	OTU0, OTU1, OTU6, OTU8, OTU9, OTU24, OTU28, OTU32, OTU49, OTU50, OTU54, OTU64, OTU66, OTU78, OTU111, OTU112, OTU113, OTU114, OTU115, OTU116, OTU117, OTU118, OTU120, OTU123	16	Uncured
**Patient7**	OTU1, OTU3, OTU6, OTU9, OTU22, OTU24, OTU67, OTU69, OTU70, OTU87, OTU89, OTU105, OTU125, OTU126, OTU128, OTU129, OTU132	OTU1, OTU3, OTU6, OTU9, OTU49, OTU64, OTU69, OTU70, OTU84, OTU87, OTU106, OTU109, OTU127, OTU128, OTU132	9	
**Patient8**	OTU0, OTU1, OTU8, OTU30, OTU32, OTU37, OTU49, OTU53, OTU54, OTU57, OTU62, OTU120, OTU122, OTU134, OTU135, OTU138, OTU139, OTU140, OTU141, OTU142, OTU144	OTU0, OTU1, OTU8, OTU24, OTU32, OTU33, OTU49, OTU53, OTU54, OTU57, OTU62, OTU64, OTU67, OTU78, OTU122, OTU134, OTU135, OTU136, OTU137, OTU139, OTU143	13	
**Patient11**	OTU3, OTU5, OTU63, OTU106, OTU108, OTU109, OTU110	OTU1, OTU3, OTU5, OTU6, OTU8, OTU9, OTU22, OTU49, OTU147	2	
**Patient15**	OTU3, OTU105, OTU109, OTU133		0	
**Patient19**	OTU0, OTU1, OTU3, OTU6, OTU32, OTU33, OTU49, OTU52, OTU54, OTU57, OTU59, OTU62, OTU64, OTU66, OTU91,		0	

	OTU143			
**Patient23**	OTU3, OTU109	OTU3	1	
**Patient25**	OTU1, OTU3, OTU6, OTU9, OTU24, OTU44, OTU51, OTU63, OTU64, OTU67, OTU69, OTU70, OTU79, OTU85, OTU105, OTU107, OTU108, OTU110, OTU127, OTU179	OTU3	1	
**Patient29**	OTU1, OTU3, OTU4, OTU6, OTU10, OTU12, OTU24, OTU43, OTU45, OTU49, OTU61, OTU63, OTU64, OTU67, OTU75, OTU76, OTU78, OTU79, OTU83, OTU84, OTU85, OTU86, OTU131, OTU145, OTU153, OTU174, OTU178, OTU186, OTU187, OTU188, OTU189, OTU190, OTU191, OTU192, OTU193, OTU194, OTU195, OTU196, OTU197, OTU198, OTU199, OTU200, OTU201, OTU202, OTU203	OTU4, OTU8, OTU186, OTU187, OTU204	3	
**Patient30**	OTU0, OTU1, OTU2, OTU7, OTU19, OTU26, OTU28, OTU32, OTU33, OTU46, OTU47, OTU49, OTU53, OTU57, OTU62, OTU93, OTU95, OTU96, OTU98, OTU99, OTU100, OTU102, OTU103, OTU104, OTU120, OTU137, OTU139, OTU205	OTU0, OTU1, OTU5, OTU7, OTU8, OTU32, OTU37, OTU49, OTU52, OTU54, OTU92, OTU134, OTU139, OTU205	7	
**Patient31**	OTU0, OTU1, OTU3, OTU8, OTU24, OTU49, OTU55, OTU64, OTU70, OTU80, OTU105, OTU119, OTU206, OTU207	OTU3, OTU105	2	
**Patient33**	OTU3	OTU3	1	
**Patient34**	OTU3, OTU109	OTU3	1	
**Patient37**	OTU0, OTU1, OTU8, OTU24, OTU54, OTU57, OTU64, OTU78, OTU121, OTU154	OTU1, OTU49	1	
